# Active and Intelligent Biodegradable Packaging Based on Anthocyanins for Preserving and Monitoring Protein-Rich Foods

**DOI:** 10.3390/foods12244491

**Published:** 2023-12-15

**Authors:** Bifen Zhu, Yu Zhong, Danfeng Wang, Yun Deng

**Affiliations:** Department of Food Science & Technology, Shanghai Jiao Tong University, 800 Dongchuan Road, Shanghai 200240, China; bifen_zhu@sjtu.edu.cn (B.Z.); zhongyu@sjtu.edu.cn (Y.Z.); llz26@sjtu.edu.cn (D.W.)

**Keywords:** food packaging, anthocyanins, biodegradable materials, food quality

## Abstract

Currently, active and intelligent packaging has been developed to solve the spoilage problem for protein-rich foods during storage, especially by adding anthocyanin extracts. In such a film system, the antioxidant and antibacterial properties were dramatically increased by adding anthocyanins. The physicochemical properties were enhanced through interactions between the active groups in the anthocyanins and reactive groups in the polymer chains. Additionally, the active and intelligent film could monitor the spoilage of protein-rich foods in response to pH changes. Therefore, this film could monitor the sensory acceptance and extend the shelf life of protein-rich foods simultaneously. In this paper, the structural and functional properties of anthocyanins, composite actions of anthocyanin extracts and biomass materials, and reinforced properties of the active and intelligent film were discussed. Additionally, the applications of this film in quality maintenance, shelf-life extension, and quality monitoring for fresh meat, aquatic products, and milk were summarized. This film, which achieves high stability and the continuous release of anthocyanins on demand, may become an underlying trend in packaging applications for protein-rich foods.

## 1. Introduction

Protein-rich foods, including fish, shrimp, meat, and dairy products, are important sources of protein, fat-soluble vitamins, micronutrients, and special flavor. Thus, protein-rich foods have a high demand and are crucial for maintaining human health. However, along the supply chain, oxidative reactions and microbial infections lead to the deterioration of the flavor, texture, and color of protein-rich foods. At the same time, ketones, aldehydes, and aromatic amines produced via the oxidation of lipids and proteins are potentially toxic. Additionally, food contamination caused by pathogenic microorganisms can easily lead to serious foodborne illness [1]. It is difficult for consumers to evaluate these changes in most cases. Meanwhile, misjudgments on food quality and safety can lead to food waste [2,3]. In order to mitigate and monitor these potential risks, appropriate packaging is needed.

Traditional plastic packaging has excellent mechanical, barrier, and thermal properties, mitigating the impact of the external environment on food. However, plastics are difficult to biodegrade and recycle, and are mainly treated via incineration and discarded in landfills, which easily pollute water, soil, and air and cause severe environmental problems. Natural materials, such as polysaccharides, proteins, and lipids are biodegradable, environmentally friendly, and readily available. Furthermore, they have good film-forming ability [4,5]. However, the functionality of single-component films is restricted. For example, pure starch film has poor water resistance and tensile strength, but good barrier properties [6]. Similarly, gelatin film possesses good physical properties but lacks antioxidant and antibacterial ability [7]. Studies have shown that the addition of natural active ingredients, in particular anthocyanin extracts, could enhance the function of biodegradable films, thereby expanding its application in protein-rich food preservation and spoilage monitoring [8,9,10].

Anthocyanins as natural pigments are safe, non-toxic, rich in resources, and are commonly used as food colorants and additives in pharmaceuticals and health products, which are also highly favored by consumers [11,12]. Previous studies have shown that anthocyanins have good color responsiveness to pH changes caused by the spoilage of protein-rich foods. Moreover, the added anthocyanins into biomass materials could improve the UV blocking capacity, reduce the oxygen and WVP of films, and inhibit the growth of microorganisms in protein-rich foods [1,13,14].

In previous reviews, the preparation, function, and application of active or intelligent packaging with natural polymers and functional additives, including anthocyanins, essential oils, and nanocomponents as the main components in food preservation have been comprehensively discussed [15,16,17]. Different from previous reviews, this article highlights active and intelligent biodegradable packaging based on anthocyanins for the preservation and monitoring of protein-rich foods. Firstly, the extraction and functional properties of anthocyanins are briefly introduced. Additionally, the mixing of anthocyanins with different bio-based materials, the improvement of anthocyanins on film function, and the on-demand release of anthocyanins via substrates are discussed. Additionally, the preservation and monitoring of active and intelligent films for meat, aquatic products, and milk are summarized. Finally, future research and the commercial prospects of active and intelligent packaging are predicted.

## 2. Extraction, Structure, and Function of Anthocyanins

### 2.1. Extraction of Anthocyanins

Solvent extraction is usually used to extract anthocyanins with ethanol, methanol, acetone, water, or mixtures of the aforementioned solvents as the medium. In addition, anthocyanins may degrade during extraction, and the stability of anthocyanins can be improved by adding formic acid or hydrochloric acid [15]. However, alcoholic extracts have poor heat stability and a short half-life. In recent years, the use of natural deep eutectic solvents (NADESs) to extract anthocyanins has become a hotspot. NADEs are present in plants and consist of natural components, such as primary metabolites. The hydroxyl or carboxyl groups in NADEs form hydrogen bonds with the extracts, which can increase the extraction rate of the target compounds. In addition, NADEs have the advantages of high chemical stability, reusability, and non-toxicity as extraction agents. They are commonly used in the extraction of polyphenols, flavonoids, proteins, and other bioactive substances [18]. Therefore, the extraction of anthocyanins using NAEDs can improve their extraction rate and stability. Bi et al. experimented with the extraction of mulberry anthocyanins using six different NADESs and found all of them exhibited higher extraction yields compared to acidified ethanol, with the best results obtained using ChCl/lactic acid [19]. Jovanović et al. developed a NADES-based extraction process for elderberry anthocyanins. The study showed that anthocyanins extracted using a ChCl/xylitol NADES exhibited the highest antioxidant and antimicrobial activities and had better stability [20]. Moreover, the acidity of the solution can affect the extraction efficiency of anthocyanins. Acid NADESs can improve the extraction efficiency of anthocyanins by preventing the degradation of non-acylated anthocyanins [21].

### 2.2. Chemical Structure and Color Indication Mechanism of Anthocyanins

**Figure 1:**
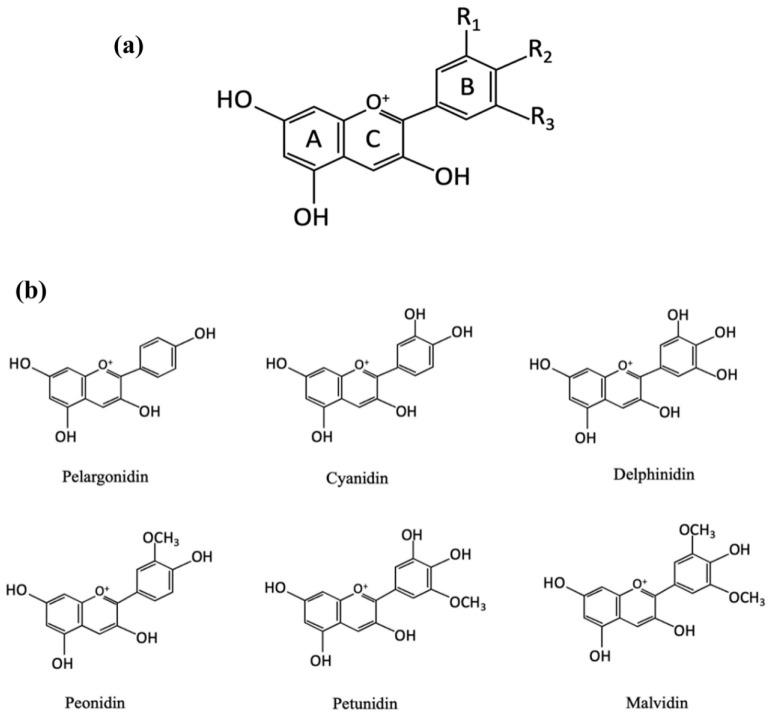
(**a**) The basic structure of a typical anthocyanin, (**b**) the chemical structures of different types of anthocyanidins. Reprinted with permission from Ref. [15]. Copyright 2022 Elsevier.

**Figure 2:**
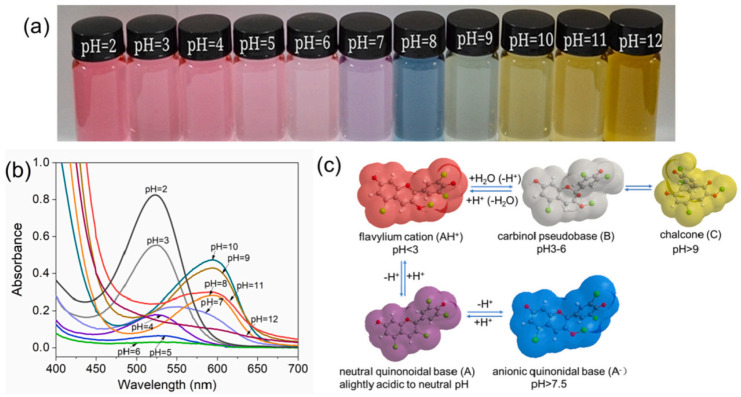
Color changes (**a**) and absorption spectra (**b**) of *Perilla frutescens* (L.) Britt. anthocyanins solutions at pH 2−12, corresponding structural transformation (**c**). Reprinted with permission from Ref. [26]. Copyright 2022 Elsevier.

Flowers, leaves, stems, and fruits show different colors due to the different types and contents of pigments, which are mainly affected by flavonoids, carotenes, and beet pigments. Anthocyanins are the most important color-developing substances in flavonoids, with C_6_-C_3_-C_6_ as the carbon skeleton and 3,5,7-trihydroxy-2-phenylbenzopyran as the basic structural unit (Figure 1a) [15,22,23]. Due to the methylation and hydroxylation of different carbon positions in anthocyanins, more than 700 anthocyanins in 27 classes have been discovered [24]. There are six common anthocyanins (Figure 1b), including pelargonidin, cyanidin, delphinidin, peonidin, petunia, and malvacin [15]. Furthermore, anthocyanins exhibit high reactivity to pH values. In H^+^ or OH^−^ environments, the distribution of π electrons in the pigment molecules changes, resulting in changes in the structure of anthocyanins. This results in changes in the absorption and reflection of light from anthocyanins, leading to displays of different colors [25,26] (Figure 2). In addition, the molecular structure of anthocyanins also affects antioxidant and antimicrobial activity [27,28]. This will be analyzed in detail in the following sections.

### 2.3. Antioxidant Mechanisms of Anthocyanins

As summarized in Table 1, anthocyanins from sources such as jambolao skins [29], black rice bran [30], shikonin [31], and blueberry [32], have scavenging effects on ABTS and DPPH. This is attributed to the conjugated structure, hydroxyl functional groups, o-diphenol structure, and polar substituents present in the molecular structure of anthocyanins, which endow them with antioxidant activity and render them inhibitors of oxidative reactions [33,34]. Moreover, it was demonstrated that the anthocyanin glycoside antioxidant was higher than glycosides, monoglycosides were higher than polysaccharides, and anthocyanosides were higher than acylated anthocyanosides [35]. Typically, anthocyanins have multiple aromatic rings that form a conjugated structure. This conjugated structure endows anthocyanins with good electron transfer properties, allowing them to capture and neutralize free radicals. In addition, the o-diphenol structure of anthocyanins enhances their ability to capture free radicals. The o-diphenol structure at the 3′ and 4′ positions on the B ring of anthocyanins forms a more stable o-quinone structure or conjugated semiquinone via two single-electron transfer reactions with RO·. Moreover, the hydroxyl functional group in anthocyanins can provide hydrogen atoms or electrons, thus stabilizing free radicals and reducing the occurrence of oxidation reactions. For example, the 5′-hydroxyl group on the A ring of anthocyanins can be easily oxidized, releasing H^+^, which has a strong scavenging effect on RO·. Then, RO· combines with 3′, 5′, and 7′ hydroxyl groups to form pseudo-semiquinone structures and undergoes keto-enol tautomerization to improve its stability [12]. Unlike other polyphenols, anthocyanins lack an electron in the C ring, forming a secondary oxonium ion, which easily attracts free radical attacks.

### 2.4. Antimicrobial Mechanisms of Anthocyanins

Table 1 summarizes the inhibitory effects of different sources of anthocyanins against common foodborne pathogens, such as *Salmonella Enteritidis*, *Pseudomonas fluorescens*, *Escherichia coli*, and *Staphylococcus aureus* [36,37,38,39]. The molecular structure of anthocyanins contains several hydroxyl functional groups, such as the hydroxyl groups in phenyl rings and aromatic alcohols. These hydroxyl groups react with lipid peroxides on microbial cell membranes, leading to membrane damage and lysis. In addition, the hydroxyl functional groups can undergo ionization and exhibit acid-base properties. Under acidic conditions, anthocyanin molecules, in the form of positively charged ions, bind to the negatively charged components (proteins and cell wall polysaccharides) of the bacterial surface. This changes the membrane potential of bacteria and disrupts the cycle of bacteria. Additionally, anthocyanins can interact with the electron transport chains in microbial cells, interfering with enzyme systems and ATP synthesis, thereby affecting microbial metabolism and proliferation. This is attributed to the existence of multiple conjugated aromatic rings in the anthocyanin structure, which endows them with electron-transferring ability. In summary, anthocyanin-microbe interactions occur through specific structural interactions between anthocyanins and microorganisms. Thus, structural differences result in different responses of microorganisms to the same anthocyanin. For example, anthocyanins may exhibit excellent inhibitory and bactericidal properties against one type of microorganism, while having no effect on another. Research has demonstrated that red onion skin extracts had an inhibitory effect on *S. aureus* DSM 20,231 but had no inhibitory effect on *E. coli* DSM 30,083 and *Salmonella* DSM 13,772 [38]. However, Sagar et al. discovered that onion extracts have antimicrobial activity against gram-negative bacteria [40]. This may be related to the onion species.

## 3. Active and Intelligent Films Containing Anthocyanins

Anthocyanins are susceptible to light, heat, humidity, and oxygen. To promote the application of anthocyanins into food packages, it is important to compound anthocyanins into a suitable matrix. Biomass materials are more sustainably sourced and less environmentally polluting than non-renewable resources. Additionally, biomass materials can effectively disperse anthocyanins and bind them through hydrogen bonding or electrostatic interactions [5]. Many studies have indicated that the incorporation of anthocyanins causes film color changes in response to conditions and improves the film’s mechanical properties, UV-blocking ability, and antibacterial and antioxidant capacities [5,10,41,42,43,44]. After a preliminary literature search, the substrates obtained directly from natural product polysaccharides (cellulose, chitosan, and starch) and proteins (gelatin) that have been used to develop active-freshness indicator packaging were summarized (Table 1).

### 3.1. Cellulose-Based Film

Cellulose is the most abundant renewable resource in the world and has a linear and high molecular weight structure. The linear arrangement of cellulose renders it a comparatively ordered crystalline structure, producing a cellulose film with good mechanical strength and stability. However, cellulose is highly crystalline in its natural state and only soluble in a few organic solvents, which renders it less susceptible to film formation [16,45,46]. Compared to natural cellulose, cellulose derivatives, such as cellulose acetate [26], methylcellulose [29], and hydroxypropyl methylcellulose [47] possess good film-forming, high modulus, and strong barrier properties, rendering them favorable for active-freshness indicator packaging. However, the hydroxyl group of cellulose is replaced by a methoxy group, resulting in the high solubility of methylcellulose. It was found that adding jambolao extracts (50%) could enhance the water resistance of methylcellulose film, which was attributed to the interaction of jambolao extracts with methylcellulose via intermolecular hydrogen bonds. In addition, these extracts could improve the mechanical properties of methylcellulose film, while decreasing the water vapor permeability [29]. Moreover, anthocyanins, as pH color indexes, endow cellulose derivative films to indicate food freshness [47,48]. You et al. fabricated a carboxymethyl cellulose/konjac glucomannan composite film that incorporated blackcurrant anthocyanins (BCAs). The composite film was red at pH 2–3, light pink at pH 4–8, and yellow-green at pH 9–13. In addition, the composite film inhibited both *E. coli* and *S. aureus* [9].

**Table 1:** Preparations of active and intelligent films that contain anthocyanins.

Substrates	Extracts	Methods	Effects of Anthocyanins	Reference
Carrageenan	Jaboticaba peels (50 and 100% *w*/*w* based on the polymer)	casting	Improves opacity, UV-vis light barrier against *E. coli*, scavenges DPPH,	[14]
Methylcellulose	Jambolao skins (0, 10, 30, and 50% based on the polymer)	casting	scavenges ABTS and DPPH, increases mechanical and barrier properties	[29]
Gelatin, oxidized chitin nanocrystals	Black rice bran (50 and 100% *w*/*w* based on the polymer)	casting	UV–vis light barrier and scavenges ABTS, DPPH, and FRAP	[30]
Potato starch	Onion (0.1% *w*/*v* based on the polymer)	casting	Action against *Staphylococcus aureus* DSM 20,231, *Salmonella bongori* DSM 13,772, and *Escherichia coli* DSM 30083, scavenges DPPH	[38]
Cassava starch	Red cabbage (7% *v*/*v* based on the polymer)	casting	Improves mechanical strength and hydrophobicity	[49]
Quercetin-loaded chitosan, agar, sodium alginate	Purple sweet potato (7% *v*/*v* based on the polymer)	casting	UV blocking and water vapor barrier	[50]
Starch, agar	Shikonin (1% *w*/*w* based on the polymer)	casting	UV-light barrier, mechanical strength, scavenges DPPH and ABTS, action against *Listeria monocytogenes*	[31]
Chitosan	Black rice bran (5, 10, and 20% *w*/*w* based on the polymer)	casting	UV–vis light barrier, sensitive and rapid response to pH/NH_3_, scavenges DPPH, reduces spoilage bacteria	[36]
Alginate, carboxymethyl chitosan	Purple cauliflower (10% *w*/*w* based on the polymer)	casting	Improves mechanical strength, reduces swelling, improves the sensitivity of the colorimetric response	[10]
Hydroxypropyl methylcellulose	Epigallocatechin-3-gallate (0.5, 1, and 2% *w*/*v* based on the polymer)	casting	Enhances mechanical strength, superior water vapor barrier, UV protection, detects bacterial growth, kills bacteria on demand	[47]
Zein	Blueberry (1, 5, and 10% *w*/*w* based on the polymer)	casting	Scavenges DPPH and ABTS and action against *E. coli* and *S. aureus*	[32]
Chitosan, cassava starch	Mulberry anthocyanin (1.7% *w*/*w* based on the polymer)	casting	Reduces oxygen and water vapor transmittance, scavenges DPPH, action against *E. coli* and *S. aureus*	[51]
Gelatin, carrageenan	Shikonin (10% *w*/*w* based on the polymer)	casting	UV blocking and action against *E. coli* and *L. monocytogenes*	[52]
Cellulose nanofiber	*Brassica oleracea* (6% *w*/*w* based on the polymer)	casting	UV blocking, improves the physicochemical properties, scavenges DPPH and ABTS,	[48]
Gelatin	Alizarin (20% *w*/*w* based on the polymer)	casting	rapid response to pH/NH_3_, light barrier, hydrophobicity, scavenges ABTS, action against *E. coli* and *S. aureus*	[23]
Cellulose acetate	*Perilla frutescens* (10% *w*/*w* based on the polymer)	electrospinning	Scavenges DPPH, enhances hydrophobicity, action against *E. coli* and *S. aureus*	[26]
Locust bean gum, polyvinyl alcohol, chitosan, *κ*-carrageenan	Purple sweet potato, Purple cabbage (1% *w*/*w* based on the polymer)	casting	Improves light barrier, scavenges DPPH, ammonia sensitivity	[8]
Potato starch	Blueberry (7.5% *w*/*w* based on the polymer)	casting	Improves mechanical properties and is ammonia-responsive	[53]
Gelatin, zein	Blueberry (5% *w*/*v* based on the polymer)	electrospinning	Fe^2+^ enhances the color response of anthocyanins	[7]
Gelatin	*Coleus scutellarioides* (10, 20, and 30% *v*/*v* based on the polymer)	casting	Increases film flexibility, decreases tensile strength, UV-vis light transmittance	[54]
Gelatin	Haskap berries (0, 0.5, 1, 2, and 3% *w*/*w* based on the polymer)	casting	increases water vapor, UV-vis light barrier, improves tensile strength, scavenges DPPH	[55]

### 3.2. Chitosan-Based Film

**Figure 3:**
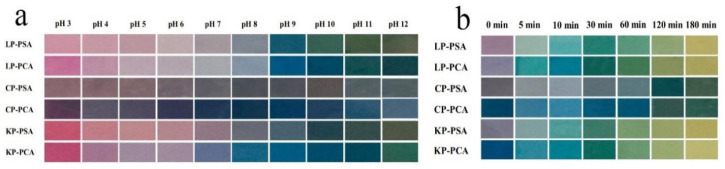
(**a**) Color changes of different polysaccharide/PVA films with PSAs or PCAs after being immersed in buffer solutions (pH 3–12) for 1 min. (**b**) Color changes of different polysaccharide/PVA films with PSAs or PCAs after being exposed to ammonia (1 mol/L) at 20 °C for 5–180 min. Reprinted with permission from Ref. [8]. Copyright 2022 Elsevier.

Chitosan, derived from deacetylated chitin, has an obvious inhibitory effect on bacteria and fungi [4,13,56,57]. The positively charged -NH^3+^ group in chitosan molecules can adsorb negatively charged bacteria, disrupting the integrity of cell walls, increasing membrane permeability, and causing the leakage of cellular contents. Inside the cell, chitosan can adsorb and bind to proteins and nucleic acids, influencing the normal physiological functions of microorganisms and inhibiting their growth and reproduction. Moreover, a large number of amino and hydroxyl groups exist on the molecular chain of chitosan and can selectively bind metal ions (such as Mg^2+^ and Ca^2+^) on the outer membrane of bacteria, inhibiting the production of bacterial toxins [10]. Furthermore, chitosan exhibits excellent film-forming properties and is transformed into highly transparent, non-toxic, and edible film. However, the mechanical properties, water resistance, and gas barrier of pure chitosan films are deficient [58]. The incorporation of anthocyanins into a chitosan film improves its mechanical properties and air barrier capacity. This is mainly due to the interaction of the hydroxyl groups of anthocyanins with the hydroxyl and amino groups of chitosan. Yong et al. developed an active/intelligent film by adding purple cabbage anthocyanins (PCAs) and purple sweet potato anthocyanins (PSAs) into chitosan/polyvinyl alcohol (CP), κ-carrageenan/polyvinyl alcohol (KP), and locust bean gum/polyvinyl alcohol (LP) matrices. The results indicated that the incorporation of PSAs and PCAs improved the film’s homogeneity, light barrier, and antioxidant, pH, and ammonia sensitivity through electrostatic interactions and hydrogen bonding between anthocyanins and the matrix. Due to the pH sensitivity of PSAs and PCAs, the films showed obvious color changes (Figure 3a) under pH values of 3–12. In addition, the color changes of PSA and PCA films in the presence of ammonia were significant (Figure 3b) [8]. Similarly, Li et al. found that pure chitosan film showed some antioxidant activity and adding mulberry anthocyanins to the film significantly improved the DPPH-free base scavenging ability [51].

Moreover, chitosan can control the release of anthocyanins, achieving the goal of extending the shelf life of protein-rich foods and the immediate indication of the edibility endpoints [36,51]. Wang et al. fabricated a chitosan/esterified chitin film loaded with eggplant peel (EE) derived anthocyanins. They found that the release rate and cumulative release of EE in different food simulation systems were different. CS demonstrated suitable solubility in acetic acid, causing its structure to be more stretched, thus allowing the rapid release of EE in 3% acetic acid. Meanwhile, the release rate of EE decreased sequentially in 50% ethanol, 10% ethanol, and distilled water, which may be due to the better solubility of EE in alcoholic solutions. In addition, attributed to the electrostatic interactions and hydrogen bonds between CS and EE, the release of total anthocyanins in all films was incomplete, which favors a sustained release effect [59].

### 3.3. Starch-Based Film

Starch has become the most promising material for the production of biodegradable polymers with low cost and good film forming [60,61,62]. The formulation of starch film is critical and determines the barrier and mechanical properties. The addition of anthocyanin extracts can prevent the cracking and brittleness of starch film, increase the flexibility and ductility of starch film, and allow starch film to demonstrate rich color changes with changes in pH value. Moreover, starch can encapsulate anthocyanins through electrostatic interactions, hydrogen bonding, and hydration, which could enhance the stability of anthocyanins [63,64]. Additionally, the branching structure of starch molecules has more voids and adsorption sites, which would enhance the encapsulation of anthocyanins. The release of anthocyanins from a film can be controlled by adjusting the shape and size of the starch molecules [65,66]. Zhang et al. compared the release of shikonin from starch and agar-based films in 50% ethanol and water. The results showed that the release of shikonin was very rapid in the first 30 min, and due to its alcohol solubility, the release of shikonin in 50% ethanol was faster. In addition, the release of shikonin from an agar-based film was faster due to the higher swelling rate of agar than that of starch [31]. Furthermore, the molecular interactions between starch and anthocyanins affect its conformation, which explains why the color changes of anthocyanins may not match the anthocyanin film, even at the same pH value [6,49,53,67].

### 3.4. Gelatin-Based Film

Gelatin is arranged with proline, hydroxyproline, and glycine repeating units [16]. The molecular structure of gelatin contains a number of hydroxyl groups, which enables it to form colloidal particles in aqueous solutions, with the cross-linking effect potentially forming a stable and flexible gelatin film with good barrier properties [7,30,52]. The formation of intermolecular hydrogen bonds between anthocyanins and gelatin significantly increases the tensile strength, WVP and UV-visible barriers, and antioxidant capacities of gelatin films [55]. Moreover, the addition of natural anthocyanins would enhance the pH-color response of the gelatin film, which could be used to maintain food quality and monitor food freshness [23,54,55]. For instance, a novel pH-sensitive and active indicator incorporated with *Coleus scutellarioides* anthocyanin extracts (CSAEs) was fabricated for fish conservation by Hematian et al. Compared with gelatin film, the film containing CSAEs had good EAB and UV light barrier capacity, but lower TS. The CSAE film was purple at an acidic pH and green at an alkaline pH [54].

### 3.5. Film-Forming Methods

For the preparation of active and intelligent biodegradable packaging, the casting method is the most commonly used, which is simple and convenient. However, starch and gelatin need to be heated to higher temperatures to form a film, while anthocyanins are easily denatured at high temperatures. This affects the color indication, antibacterial, and antioxidant effects of anthocyanins [31,49]. Electrostatic spinning can continuously prepare sub-micron or nano-sized ultrafine films without high temperatures and pressure. Moreover, films formed via electrospinning have unique pore structures, large specific surface areas, and easily modified surfaces, which provide significant advantages in stimulus sensing and the controlled release of anthocyanins [7,26]. In addition, three-dimensional (3D) printing can quickly and accurately print composite labels with specified shapes, avoiding shape and size errors, and each film can be loaded with the same amount of anthocyanins [51]. Li et al. found that 3D printing placed anthocyanins in the right position within the indicator film, which could reduce the over-oxidation of anthocyanins without affecting their antimicrobial, antioxidant, and color responses [51].

## 4. Application in Protein-Rich Foods

### 4.1. Fresh Meat

**Figure 4:**
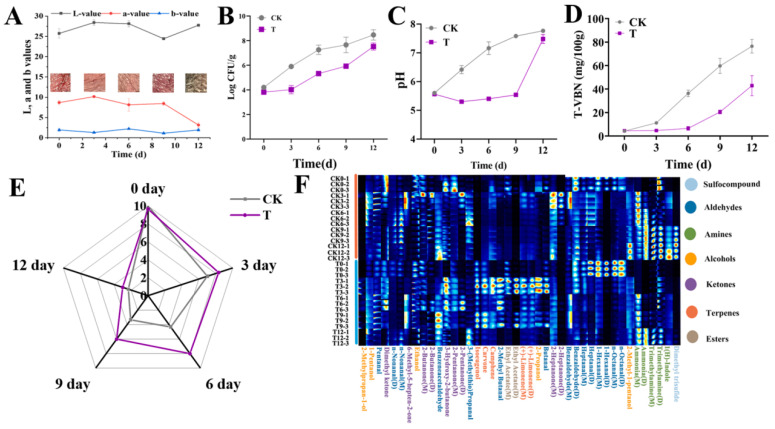
The color values (**A**), total viable counts (**B**), pH values (**C**), TVB-N values (**D**), changes in sensory indexes (**E**), and fingerprints of volatile compounds (**F**) of pork samples during storage at 4 °C. CK: the pork with the CS-OEO-BRBA film, T: the pork without a film wrap. Reprinted with permission from Ref. [36]. Copyright 2022 Elsevier.

Fresh meat is prone to lipid peroxidation and microbial contamination during processing, transportation, storage, and consumption. This would result in color, odor, and pH changes which impact the acceptability of the meat product [68]. For these reasons, fresh meat has a very short shelf life at 4 °C (3–5 days) [69]. Active and intelligent films containing anthocyanins could delay meat spoilage and indicate the freshness of meat [70]. For example, Hao et al. prepared a CS-OEO-BRBA film using chitosan embedded with black rice bran anthocyanins (BRBAs) and oregano essential oil (OEO). The CS-OEO-BRBA film could improve the sensory and color quality indexes of pork, slow down the rise of pH, and reduce the TVB-N value at 4 °C (Figure 4). The inclusion of BRBA and OEO reduced the abundance of spoilage bacteria in pork and delayed the emergence of odor volatiles. Moreover, the CS-OEO-BRBA film turned bottle-green on day 12 (with a red color at the beginning), indicating that the pork had lost its commercial value [36]. Wang et al. developed an active and intelligent film to monitor the freshness of pork. The TVB-N value of the pork on the first day was 15.16 ± 1.15 mg/100 g, which indicated that the pork was not fit for consumption, but the change in the appearance of the pork was difficult to observe with the naked eye. However, the color of the film had changed from blue to navy blue. On the second day, the color of the film changed to green and the TVB-N value of the pork had increased to 24.32 ± 1.02 mg/100 g, which indicated the pork had been severely spoiled [59].

Moreover, the antioxidant and antibacterial activity of anthocyanins could also extend the meat’s shelf life. The addition of an *Amaranthus* leaf extract (ALE) significantly delayed the growth of the total bacterial count (TBC) and *S. aureus* during chicken preservation when chilled. *S. aureus* and the TBC increased to 3.88 log CFU/g and 6.53 log CFU/g on day 3 in the control group, while the ALE film-packaged group increased to 2.91 log CFU/g and 6.00 log CFU/g after 12 days. At the same time, the film changed from red to yellow when the chicken transitioned from fresh to rotten [71]. Liu et al. showed that adding butterfly bean anthocyanins could extend the freshness of beef stored at 4 °C for two days. When the film’s color changed from purple-blue to blue-green, the beef changed from fresh to sub-fresh and then corrupt [72].

### 4.2. Aquatic Products

Aquatic products are loved by consumers, but during storage after deactivation, microbial growth and enzymatic reactions trigger biochemical reactions, leading to spoilage. It not only causes the loss of the eating quality and nutritional value of aquatic products but also brings food safety problems [50]. The bacteria-induced spoilage of aquatic products produces basic compounds resulting from the degradation of proteins, such as trimethylamine and dimethylamine, and the consequent pH increase is considered to be an important indicator of quality deterioration [43]. Changes in pH values can alter the molecular structure or conformation of the anthocyanins within the active and intelligent film, inducing a change in the film color to determine the quality of the aquatic products [54,68]. In conclusion, the color of films is highly correlated with the sensory quality, lipid peroxide, colony count, volatile salt nitrogen, and pH value of the food.

Ezati et al. observed that the controlled release of shikonin from complex films exhibited strong antioxidants (ABTS and DPPH). In addition, the film supplemented with shikonin had an obvious inhibitory effect on *L. monocytogenes*. After 12 h, the growth rate of *L. monocytogenes* was 3-fold lower in the shikonin-added films compared to the pure film. In particular, the complex films showed quick color changes when exposed to ammonia vapor and different pH values. The complex films showed a characteristic color change from reddish-pink to bluish-purple when used for shrimp packaging, indicating the onset of shrimp spoilage [31]. Wu et al. also found that after fresh shrimp were stored at 25 ℃ for 24 h, the *Clitoria ternatea* extract-added film changed from blue to blue-green, indicating that the shrimp changed from fresh to rotten [73]. Moreover, Kanatt developed an *Amaranthus* leaf extract-added film, which could effectively reduce the total bacterial count of fish stored at 4 °C and inhibit the growth of *S. aureus* and oxidative rancidity. This increased the shelf life of fish from three days to twelve days. When the film color changes from red to yellow, it means that the fish is no longer edible [71].

### 4.3. Milk

Milk, as a nutritious and comprehensive food, contains high-quality proteins, oligosaccharides, fats, and vitamins. It is also highly susceptible to the growth of microorganisms, carbohydrate fermentation, fatty acid failure, and protein denaturation, thus spoiling nutrition. A significant amount of milk is wasted due to spoilage during distribution and consumption. Milk stored in supermarkets or at home must be checked for freshness before consumption. Currently, the commonly used methods to monitor milk freshness are nuclear magnetic resonance, near-infrared spectroscopy, and mid-infrared spectroscopy, which need expensive equipment and tedious operations [7]. In this regard, active and intelligent packaging has emerged to provide convenience, the real-time monitoring of food safety and quality, and reduce food waste. Carrageenan/gelatin-based films containing shikonin exhibited terrific activity against *L. monocytogenes* and *E. coli*. The film showed pH-dependent color variation; red at pH 2–7, purple at pH 9, and blue at pH 10–12. After three months, the film still exhibited good color stability. The film changed from purple to reddish-pink when it is immersed in fresh, decaying, and spoiled milk for 10 min. At the same time, the red chromatic shift index, pH, and degree of spoilage of milk corresponded with each other [52]. Additionally, Gao et al. prepared an indicator film for monitoring milk freshness by incorporating gelatin, blueberry anthocyanins, and Fe^2+^ into a corn protein matrix using the electrostatic spinning method. The change in the film color was visually perceptible from purple-black (fresh milk), royal purple (spoiled milk), to purple-red (spoiled milk). At the same time, the color parameters of the film (L*, a*, R, G, and B) were highly correlated with the pH of the milk during storage [7]. Moreover, the freshness monitor could reflect the digitized color information via an intelligent phone [50].

## 5. Summary and Future Prospects

In recent years, the environmental pollution caused by petroleum plastics and the growing consumer demand for convenient and fresh foods has promoted the development of new food packaging options. Active and intelligent films based on natural anthocyanins can monitor the real-time freshness of food and extend the shelf life of food. This paper reviewed the extraction, structure, and function of anthocyanins, and summarized the types of active and intelligent films and their applications in protein-rich foods. The results showed that anthocyanins could improve the mechanical properties and barrier properties of the substrate. In addition, anthocyanins have antimicrobial, antioxidant, and unique pH-responsive color changes. As an easy-to-use film for food freshness retention and monitoring, active and intelligent films have great commercial potential in the packaging of protein-rich foods. Moreover, active and intelligent films based on natural anthocyanins are still a long way from commercial applications. For example, highly stable anthocyanins with definite structures need to be explored; film manufacturing processes suitable for commercial mass production are still under investigation; and the color changes caused by the sensitivity, stability, and reproducibility of active and intelligent films need to be more systematically researched. Hence, convenient and sensitive active and intelligent films should be developed for fresh-cut fruits and vegetables, meat, fish, and milk that reduce food waste and health problems caused by food quality and safety.

## Data Availability

Data is contained within the article.
